# Phenolics and Antioxidant Activity of Mulberry Leaves Depend on Cultivar and Harvest Month in Southern China

**DOI:** 10.3390/ijms131216544

**Published:** 2012-12-05

**Authors:** Yuxiao Zou, Shentai Liao, Weizhi Shen, Fan Liu, Cuiming Tang, Chung-Yen Oliver Chen, Yuanming Sun

**Affiliations:** 1 College of Food Science, South China Agricultural University, Guangzhou 510642, China; E-Mail: yuxiaozou@163.com; 2 Sericulture & Agri-Food Research Institute, Guangdong Academy of Agricultural Sciences, No. 133 Yiheng ST Dongguanzhuang RD, Guangzhou 510610, China; E-Mails: liaost@163.com (S.L.); skyforce12@163.com (W.S.); liufan1234@gmail.com (F.L.); tangcuiming@163.com (C.T.); 3 Antioxidants Research Laboratory, Jean Mayer USDA Human Nutrition Research Center on Aging, Tufts University, Boston, 711 Washington St., Boston, MA 02111, USA; E-Mail: oliver.chen@tufts.edu

**Keywords:** phenolics, antioxidant activity, mulberry leaf, cultivar, harvest month

## Abstract

To elucidate the effects of cultivar and harvest month on the phenolic content and antioxidant activity of mulberry leaves, four major phenolics, including chlorogenic acid (ChA), benzoic acid (BeA), rutin (Rut) and astragalin (Ast), were quantified using an HPLC-UV method. Leaves from six mulberry cultivars, collected from April to October, were analyzed. The antioxidant activity of mulberry leaves was assessed by ferric reducing antioxidant power (FRAP), hydroxyl radical scavenging activity (HSA) and superoxide radical scavenging activity (SSA) assays. The results showed that the total values of the four phenolic compounds ranged from 2.3 dry weight (DW) to 4.2 mg/g DW, with ChA being the major compound. The mean total phenol (TP) content of the six cultivars ranged from 30.4 equivalents (GAE) mg/g DW to 44.7 GAE mg/g DW. Mulberry leaves harvested in May had the highest TP content. Moreover, the antioxidant activities of mulberry leaves harvested from April to October differed noticeably. In general, Kq 10 and May were considered to be a better cultivar and harvest month concerning phenolic content and antioxidant activity, respectively.

## 1. Introduction

China is the main producer of mulberry (genus *Morus*), an economically important plant used for sericulture. Southern China is the major mulberry growing region in the country, accounting for 30% of the planted area. The warm and humid climate of the region allows the mulberry trees to grow continuously for seven months of the year [1].

Mulberry leaves have been used in China for hundreds of years to treat hyperglycemia, inflammation, cough, hypertension, cancer and fever [2,3]. Phenolics with strong antioxidant activity toward reactive oxygen species (ROS) are thought to contribute to the biological activities of the leaves [4,5]. Although some phenolics, such as rutin, quercetin and isoquercetin, as well as some flavonoids, have been identified in mulberry leaves [6], there is currently little information on the influence of cultivar and harvest month on phenolic content and antioxidant activity. Our previous study showed that cultivar and harvest month had a significant effect on the anthocyanin content, some other phenolic compounds and antioxidant activity in mulberries [7,8]. However, further research is needed. Hence, this work attempts to understand whether the bio-efficacy conferred by phenolics to mulberry leaves is consistent with different cultivars and harvest months [6,9,10].

Therefore, we investigated the content of four major phenolic compounds (chlorogenic acid, benzoic acid, rutin and astragalin), as well as the antioxidant activity in mulberry leaves, in order to determine the influence of cultivar and harvest month on these variables.

## 2. Results and Discussion

It is known that genetics, environment and agricultural practices greatly affect the phytochemical profiles of plants; however, there is limited evidence of the impact of these factors on the bioactivities of mulberry leaves. Such information would help farmers to select cultivar(s) and a harvest time to produce mulberry leaves and related products with the greatest levels of compounds with antioxidant, anti-inflammatory and anti-diabetic activities.

### 2.1. Effect of Cultivar on Phenolics and Antioxidant Activity of Mulberry Leaves

The mean contents of four phenols (ChA, BeA, Rut and Ast) in the leaf extract of six mulberry cultivars, harvested over seven consecutive months, ranged from 2.32 mg/g DW to 4.17 mg/g DW. In particular, ChA was the major compound, representing over 44.9% of the total, while Rut, Ast and BeA were relatively low (Table 1). This result was in agreement with previous reports on Japanese mulberry leaves by Onogi *et al*. [10], Katsube *et al*. [11] and Matsuoka *et al*. [9]. Moreover, the data showed that the Rut content (1.20 ± 0.4 mg/g DW) in Kq 10 and the BeA content (0.8 ± 0.2 mg/g DW) in Ys 10 were significantly higher than other cultivars. Ys 10 had relatively high levels of the four phenolic compounds with 2.1 ± 0.6 mg/g DW ChA, 0.7 ± 0.2 mg/g DW Rut, 0.8 ± 0.2 mg/g DW BeA and 0.6 ± 0.2 mg/g DW Ast, while Kq 10 possessed relatively high level of Rut (1.2 ± 0.4 mg/g DW) and ChA (2.2 ± 0.6 mg/g DW). The profiles of the four quantified phenols were different among six cultivars and consistent with the study by Song *et al*. [12]. Conversely, Da 10 had lower levels of the four phenolic compounds compared with the other cultivars, which was in disagreement with the results of Song *et al*. [12], who suggested it to be the most valuable mulberry cultivar based on its high content of functional components.

The total phenol (TP) values in six mulberry cultivars ranged from 30.4 GAE mg/g DW to 44.7 GAE mg/g DW, with the highest TP in Kq 10 (44.7 ± 10.7 GAE mg/g DW) and the lowest in Ns 14 (30.4 ± 6.3 GAE mg/g DW) (Table 2). These results were in agreement with those reported by Jia *et al*. [6], who described a wide variation in the flavonoid contents of the leaves of 13 mulberry varieties, ranging from 9.8 mg/g DW to 29.6 mg/g DW Rut equivalents. The hydroxyl radical scavenging activity (HSA) and superoxide radical scavenging activity (SSA) values did fluctuate among six cultivars, with the highest HSA and SSA in Kq 10 and the lowest in Ns 14. However, ferric reducing antioxidant power (FRAP) values did not vary a lot. In general, the antioxidant activities of the leaves from six mulberry cultivars varied considerably.

**Figure 1:**
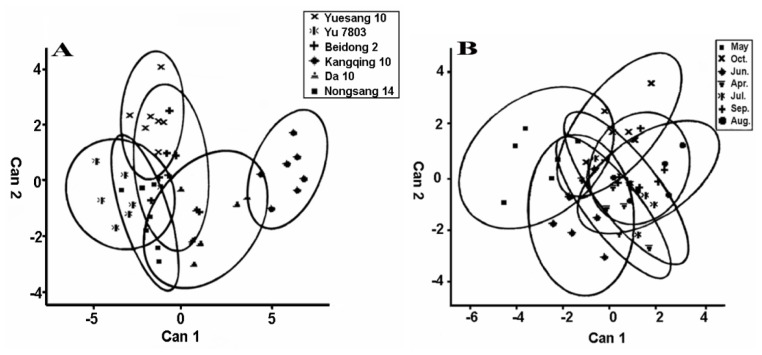
Canonical discriminant analysis of the mulberry cultivars (**A**) and harvest month of mulberry leaves (**B**).

Canonical discriminant analysis showed an obvious variability among the six cultivars, especially between Kq 10 and the other five cultivars (Figure 1A). Kq 10 only possess a significantly higher content of Rut, which implies it probably plays a very important role in the antioxidant activity of mulberry leaf. Differences among the mulberry cultivars could further explain why the corresponding leaf samples had significant differences on their phenolic content and antioxidant activity. Canonical discriminant analysis has also been used to differentiate cultivars and fertilization types in wines [13,14], olives [15], grapes [16] and almonds [17].

Further experiments showed that the TP content was significantly correlated with FRAP (*R*^2^ = 0.66), HSA (*R*^2^ = 0.75) and SSA (*R*^2^ = 0.56) (*p* ≤ 0.01) (Table 3). The results were consistent with widely accepted knowledge that phenolics contribute to the antioxidant activity of plants. Isabelle *et al*. [18] reported strong correlations between radical scavenging capacities and the content of phenolic compounds in mulberry fruits. A similar result was reported by Ozgen *et al*. [19]. Furthermore, Katsube *et al*. [20] indicated that flavonoids in mulberry leaves were the main antioxidants protecting LDL against *in vitro* copper-induced oxidation. The correlation results showed that ChA and Rut contributed to the antioxidant activity of mulberry leaves, as determined by FRAP and HSA.

It is known that both genotype and growing environment can affect phytochemical production in an interactive manner. Since all the samples were collected from the same orchard, the differences could not be ascribed to growing location, environment or agricultural practice. Thus, only the cultivar difference had an impact on the phenolic content and antioxidant activity, rather than the growing environment (Supplementary Table S1).

### 2.2. Effect of Harvest Month on Phenolics and Antioxidant Activity of Mulberry Leaves

Leaves from six mulberry cultivars, collected from April to October in southern China, were used to investigate the influence of harvest month on phenolics and antioxidant activity. ChA was the major phenolic compound and its concentration did not change significantly over seven harvest months. A similar trend was observed for BeA and Rut. However, the content of Ast was markedly affected by harvest month, with its concentration reaching the highest level in August (Table 4). Further canonical discriminant analysis showed that the effect of harvest month was less obvious than that of cultivar (Figure 1B), which could further explain the above result.

The mulberry tree is a fast-growing deciduous plant that can grow under different climatic conditions (*i.e.*, tropical, subtropical and temperate) [21]. The *Compendium of Materia Medica*, a pharmaceutical text written by Li Shi-Zhen during the Ming Dynasty, indicated that mulberry leaves harvested before natural defoliation in autumn (October) were the best for medicinal use, but no explanation was provided. Guan *et al*. [22] also indicated that mulberry leaves harvested in autumn would exert larger bio-efficacy than those harvested in summer, probably because of the higher rutin content of the former. In general, the growing period of mulberry plants is four-to-seven months, dependent on climate condition and cultivar. Jia *et al*. [6] reported that the flavonoid contents of mulberry leaves collected in spring and autumn were different. Nevertheless, our study is the first to monitor the changes in phenolic contents and antioxidant activities in mulberry leaves over seven months of the growing season. Our results showed that mulberry leaves harvested in May contained more TP and had a stronger antioxidant activity than those collected in April. However, there were differences in the levels of the four phenolic compounds during the growing season. The contents of ChA and BeA were unaltered during the whole growing season, but the contents of Rut and Ast increased continuously. However, the most bioactive constituents in mulberry leaves, responsible for their putative health benefits, remain to be investigated. The higher contents of TP and ChA, as well as the stronger antioxidant activity observed in the early harvest season, suggest that mulberry leaves collected in May are the best for medicinal use, which disagrees with both the recommendation given in the *Compendium of Materia Medica* and the study by Jia *et al*. [6].

This study also showed that the harvest month significantly affected the TP content and the FRAP and SSA activities, but not the HSA activity. After reaching their highest levels in May, TP, FRAP and SSA declined in later harvest months (Table 5).

## 3. Experimental Section

### 3.1. Materials

ChA, Rut (quercetin-3-*O*-rutinoside), BeA and Ast (kaempferol-3-*O*-glucoside) were purchased from the National Institutes for Food and Drug Control (Beijing, China). All other chemicals and reagents were from Guangzhou Chemical Reagent Factory (Guangzhou, China).

### 3.2. Mulberry Leaf Samples

Tang 10 (T 10), Kangqing 10 (Kq 10), Beidong 2 (Bd 2), Yu 7803, Yuesang 10 (Ys 10) and Nongsang 14 (Ns 14) are six typical cultivars widely grown in southern China. All the mulberry plants for this study were cultivated in an experimental field in Guangzhou, which was managed by the Sericulture & Agri-Food Research Institute of the Guangdong Academy of Agricultural Sciences. Leaves from each mulberry cultivar were harvested from the same trees once a month over seven consecutive months of the growing period (April to October) in 2009. A total of 42 mulberry leaf samples were collected. The specimen identities were confirmed by Cui-Ming Tang, a mulberry taxonomist from the Sericulture & Agri-Food Research Institute. The mulberry leaves were shade dried, powdered using an electric grinder and stored at −20 °C until extraction.

### 3.3. Mulberry Leaf Extraction

Phenols in mulberry leaf powder were extracted using 70% ethanol in water (pH 4), according to our previously published protocol [23]. Briefly, 1 g of mulberry leaf powder was mixed with 40 mL of acidified ethanol solution and then extracted using sonication for 30 min at room temperature. After centrifugation, the solvent was removed using a rotavapor over a water bath below 40 °C. The dried extracts were stored at −20 °C. Before conducting the assays, the mulberry leaf extract (MLE) was reconstituted with 25 mL of distilled water to produce a concentration equivalent to 40 mg/mL of mulberry leaf powder.

### 3.4. Determination of Four Phenolic Compounds by HPLC

Thirteen phenolic compounds from mulberry leaves were previously quantified in our laboratory using HPLC. Among them, chlorogenic acid, benzoic acid, rutin and astragalin were the predominant compounds, accounting for 76.4%–88.3% of the total (Supplementary Table S2). Some individual compounds, such as Cat, VaA, CaA, GaA, Hyp and Que, were not present in all the cultivars. Moreover, detection and quantification of Rut is the only detective index required for the quality control of mulberry leaf [3]. Therefore, four main phenols, including ChA, Rut, BeA and Ast, were quantified using an Agilent 1200 series HPLC (Agilent Technologies Inc., Karlsruhe, Germany) equipped with a Zorbax SB-C18 column (250 mm × 4.6 mm i.d., 5 μm) and an Agilent photodiode array detector. The mobile phase was 0.4% acetic acid (MPA): acetonitrile (MPB), pumped at 1 mL/min in a linear gradient mode, with 5%–25% MPB (0–40 min), 25%–35% MPB (40–45 min), 35%–50% MPB (45–50 min), and 50%–55% MPB (50–55 min). The column was kept at 30 °C, and the injection volume was 20 μL. The phenolic compounds were monitored at 280 nm, and their concentrations were calculated based on the standard curves constructed with authentic standards. Concentration was expressed as mg/g DW.

### 3.5. Total Phenols in MLE

Total phenols (TP) in water-reconstituted mulberry leaf extract (MLE) were determined by the method of Chen *et al*. [24], and the results were expressed as gallic acid equivalents (GAE) mg/g DW.

### 3.6. FRAP Assay

The FRAP value of water-reconstituted MLE was determined via a redox-linked reduction of Fe^3+^-2,4,6-tripyridyl-*S*-triazine to a blue-colored Fe^2+^ complex at pH 3.5, according to the method of Chen *et al*. [24]. MLE was incubated with the FRAP reagent for 1 h at room temperature. The absorbance was measured at 593 nm, using a Shimadzu UV1700 spectrophotometer (Shimadzu Instruments Manufacturing, CO., LTD, Suzhou, China). The FRAP value was expressed as mmol Trolox equivalents (TE)/100 g DW.

### 3.7. Superoxide Radical Scavenging Activity (SSA)

SSA was measured in a xanthine/xanthine oxidase system with spectrophotometric determination of the reduction product of nitroblue tetrazolium (NBT), according to the method described by Chun *et al*. [25], with slight modifications. Briefly, water-reconstituted MLE was incubated with a reaction mixture of 50 μmol/L NBT, 50 μmol/L xanthine and 0.05 U/mL xanthine oxidase (final concentrations) for 10 min at room temperature, and then, the change in absorbance of NBT was measured at 560 nm using a Shimadzu UV1700 spectrophotometer. The SSA was expressed as a percentage of the control, which did not contain antioxidants, based on the following equation:

(1) $$\text{Scavenging activity }(\%)=(1-A{\text{sample}}_{560}/A{\text{control}}_{560})\times 100\%$$

### 3.8. Hydroxyl Radical Scavenging Activity (HSA)

HSA of water-reconstituted MLE was assessed using the hydroxyl radical system generated by the Fenton reaction, according to the method of Heo *et al*. [26] with minor modifications. Briefly, 1 mL of water-reconstituted MLE was added to a reaction mixture of 1 mL brilliant green (0.435 mmol/L), 0.5 mL FeSO_4_ (2 mmol/L) and 1.5 mL H_2_O_2_ (3%). After 20 min of incubation at room temperature, the absorbance was measured at 624 nm, using a Shimadzu UV1700 spectrophotometer. The HSA was expressed as percentage of the control, which did not contain antioxidants, based on the following equation:

(2) $$\text{Scavenging activity }(\%)=(1-A{\text{sample}}_{624}/A{\text{control}}_{624})\times 100\%$$

### 3.9. Statistics and Data Analysis

Statistical analysis was performed using SPSS (version 17, SPSS Inc., Chicago, IL, USA, 2008). The results were expressed as mean ± standard deviation (*SD*). One-way ANOVA was performed to determine the effect of cultivar or harvest month, followed by the Tukey-Kramer HSD test, with which the significance was obtained. A *p*-value ≤ 0.05 was considered statistically significant. The Pearson’s correlation was performed between all study outcomes. Canonical discriminant analysis (CDA) was performed using SAS (version 9.1.3, SAS Institute Inc., Cary, NC, USA, 2002) with standard pooled variance and 80% confidence ellipses. Cultivar and harvest month were independently modeled using ChA, Rut, BeA, Ast, total phenol, FRAP, HSA and SSA.

## 4. Conclusions

There were significant variations in the phenolic content and antioxidant activity of leaves from six mulberry cultivars, collected over seven months. As all mulberry plants were cultivated in the same orchard, the differences could not be ascribed to growing location, environment or agricultural practice, but to genotype and maturation of leaves. Our data suggest that Kq 10 and May are the better cultivar and harvest month, respectively, in the context of phenolic content and antioxidant activity. Our results can help farmers to select a cultivar and harvest month that would allow them to produce mulberry leaves with superior quality. More work is needed to examine potential differences in the bio-efficacy of mulberry leaves from different cultivars and harvest months. Furthermore, more information is needed on the impact of processing and storage on the bioactivities of mulberry leaves.

## Supplementary Information



## Figures and Tables

**Table 1:** Effect of cultivar on the content of phenolic compounds in mulberry leaves.

Cultivar	Individual phenolic compound (mg/g DW)				Sum

	ChA	Rut	BeA	Ast	
Da 10	1.5 ± 0.6	0.4 ± 0.3 ^a^	0.1 ± 0.1 ^a^	0.3 ± 0.2	2.3 ± 1.0 ^a^
Kq 10	2.2 ± 0.6	1.2 ± 0.4 ^b^	0.3 ± 0.2 ^a^	0.3 ± 0.2	4.0 ± 0.6 ^b^
Ns 14	2.0 ± 0.3	0.2 ± 0.1 ^a^	0.5 ± 0.2 ^a,b^	0.3 ± 0.2	3.0 ± 0.7 ^a^
Yu 7803	1.6 ± 0.4	0.1 ± 0.2 ^a^	0.5 ± 0.1 ^a^	0.5 ± 0.2	2.7 ± 0.5 ^a^
Ys 10	2.1 ± 0.6	0.7 ± 0.2 ^a,b^	0.8 ± 0.2 ^b^	0.6 ± 0.2	4.2 ± 1.1 ^b^
Bd 2	2.3 ± 0.4	0.4 ± 0.2 ^a^	0.5 ± 0.2 ^a,b^	0.5 ± 0.2	3.7± 0.6 ^a,b^

The experimental results were analyzed by one-way ANOVA and Tukey-Kramer HSD. Values (mean ± SD, *n* = 7) with no letters in common are significantly different (*p* < 0.05) within a column.

**Table 2:** Effect of cultivar on TP content and antioxidant activity of mulberry leaves.

Cultivar	TP (GAE mg/g DW)	FRAP (mmol TE/100 g DW)	HSA (%)	SSA (%)
Da 10	35.4 ± 4.7 ^a,b^	11.5 ± 2.2	60.11 ± 5.9 ^b^	44.1 ± 7.1 ^a,b^
Kq 10	44.7 ± 10.7 ^b^	14.3 ± 1.5	72.8 ± 6.8 ^c^	58.3 ± 13.7 ^b^
Ns 14	30.4 ± 6.3 ^a^	12.1 ± 1.6	50.9 ± 8.6 ^a^	38.0 ± 4.6 ^a^
Yu 7803	40.2 ± 6.1 ^a,b^	13.4 ± 1.4	60.0 ± 9.3 ^b^	45.93 ± 9.5 ^a,b^
Ys 10	43.0 ± 5.7 ^b^	12.1 ± 2.5	69.3 ± 7.8 ^c^	42.2 ± 7.7 ^a^
Bd 2	44.5 ± 8.0 ^b^	13.8 ± 1.8	69.7 ± 3.3 ^c^	53.1 ± 11.4 ^a,b^

The experimental results were analyzed by one-way ANOVA and Tukey-Kramer HSD. Values (mean ± SD, *n* = 7) with no letters in common are significantly different (*p* < 0.05) within a column.

**Table 3:** Coefficients of Pearson’s correlation test between experimental parameters.

	FRAP	SASC	HASC	ChA	Rut	BeA	Ast
TP	0.66 **	0.56 **	0.75 **	0.37 *	0.41 **	0.25	0.42 **
FRAP	-	0.41 **	0.42 **	0.48 **	0.32 *	0.20	0.30
SSA	-	-	0.38 *	0.23	0.28	−0.18	0.12
HSA	-	-	-	0.35 *	0.52 **	0.13	0.29
ChA	-	-	-	-	0.28	0.34 *	0.23
Rut	-	-	-	-	-	0.03	0.17
BeA	-	-	-	-	-	-	0.57 **

Differences were considered to be statistically significant when * *p* ≤ 0.05 and ** *p* ≤ 0.01.

**Table 4:** Effect of harvest month on 4 phenolic compounds in mulberry leaves.

Harvest month	Individual phenolic compound (mg/g DW)				

	ChA	Rut	BeA	Ast	Sum
Apr.	1.9 ± 0.6	0.4 ± 0.4	0.4 ± 0.3	0.2 ± 0.2 ^a^	2.8 ± 1.3
May	2.3 ± 0.7	0.5 ± 0.4	0.6 ± 0.4	0.4 ± 0.1 ^a,b^	3.7 ± 1.1
Jun.	2.2 ± 0.4	0.5 ± 0.3	0.5 ± 0.3	0.3 ± 0.1 ^a,b^	3.4 ± 0.6
Jul.	1.7 ± 0.3	0.3 ± 0.3	0.4 ± 0.2	0.4 ± 0.1 ^a,b^	2.9 ± 0.6
Aug.	1.7 ± 0.5	0.6 ± 0.6	0.5 ± 0.2	0.5 ± 0.1 ^b^	3.3 ± 0.6
Sep.	2.0 ± 0.5	0.6 ± 0.5	0.5 ± 0.3	0.5 ± 0.3 ^a,b^	3.6 ± 1.1
Oct.	1.9 ± 0.7	0. 7 ± 0.6	0.4 ± 0.3	0.5 ± 0.3 ^a,b^	3.6 ± 1.6

The experimental results were analyzed by one-way ANOVA and Tukey-Kramer HSD. Values (mean ± SD, *n* = 6) with no letters in common are significantly different (*p* < 0.05) within a column.

**Table 5:** Effect of harvest month on TP and antioxidant activity of mulberry leaves.

Harvest Month	TP (GAE mg/g DW)	FRAP (mmol TE /100 g DW)	HSA (%)	SSA (%)
Apr.	28.7 ± 6.3 ^a^	11.2 ± 1.3 ^a^	56.4 ± 9.8	34.2 ± 4.8 ^a^
May	49.0 ± 9.0 ^b^	15.8 ± 1.1 ^b^	70.4 ± 10.8	53.8 ± 16.2 ^b^
Jun.	42.3 ± 8.4 ^b^	12.5 ± 0.9 ^a^	64.9 ± 7.7	54.1 ± 11.5 ^b^
Jul.	38.2 ± 5.1 ^a,b^	11.9 ± 2.4 ^a^	62.4 ± 11.1	48.7 ± 8.6 ^a,b^
Aug.	37.2 ± 3.5 ^a,b^	13.1 ± 1.1 ^a,b^	61.2 ± 8.7	49.7 ± 7.1 ^a,b^
Sep.	38.9 ± 5.2 ^a,b^	12.4 ± 2.4 ^a^	64.3 ± 13.9	45.3 ± 8.0 ^a,b^
Oct.	43.7 ± 7.5 ^b^	13.1 ± 1.7 ^a,b^	67.0 ± 7.0	42.8 ± 9.2 ^a,b^

The experimental results were analyzed by one-way ANOVA and Tukey-Kramer HSD. Values (mean ± SD, *n* = 6) with no letters in common are significantly different (*p* < 0.05) within a column.
