# Supplementary material for: Phenolics and Antioxidant Activity of Mulberry Leaves Depend on Cultivar and Harvest Month in Southern China
Source: Int J Mol Sci. 2012 Dec 5;13(12):16544–53. doi: 10.3390/ijms131216544 (PMC3546706; doi:10.3390/ijms131216544)
Supplement: Supplementary file 1 [file ijms-13-16544-s001.pdf]

Supplementary Information

Table S1. Species and ploid of studied mulberry leaves.

**Sample**	**Species**	**Ploid**	**Characteristics**
Da 10	*Morus. atropurpurea* Roxb.	triploid	High productivity for both leaf and seedless fruit
Kangqing 10	*Morus. atropurpurea* Roxb.	diploid	High resistance against *Pseudomonas solanacearum*
Yuesang 10	*Morus. atropurpurea* Roxb.	tetraploid	Particular high productivity of leaf
Nongsang 14	*M. multicaulis* Perr.	diploid	High productivity of leaf and high resistance against to *Mycoplasma*-like organism
Yu 7803	*M. alba* Linn.	diploid	High productivity of leaf and high resistance against to *Pseudomonas syringae pv.* mori
Beidong 2	*M. australis* Poir.	diploid	High productivity of leaf

Table S2. Effect of cultivar on the content of phenolic compounds in mulberry leaves.

**Cultivar**	**Individual Phenolic Compound (mg/g DW)**														
	**ChA**	**Rut**	**BeA**	**Ast**	**GeA**	**Cat**	**VaA**	**CaA**	**SyA**	**Epi**	**GaA**	**Hyp**	**Que**	**Sum**	**Percent of the four main individual phenolic compounds (100%)**
Da 10	1.5 ± 0.6 [a]	0.4 ± 0.3 [a,b]	0.1 ± 0.1 [a]	0.3 ± 0.2 [a]	0.1 ± 0.02 [a]	0.06 ± 0.005 [c]	0.02 ± 0.005 [a]	-	0.1 ± 0.06 [a,b]	0.3 ± 0.04 [b]	0.01 ± 0.005	0.07 ± 0.05	0.05 ± 0.04	3.01	76.4
Kq 10	2.2 ± 0.6 [a]	1.2 ± 0.4 [c]	0.3 ± 0.2 [a,b]	0.3 ± 0.2 [a,b]	0.1 ± 0.03 [a]	-	0.05 ± 0.006 [b]	-	0.1 ± 0.06 [a,b]	0.2 ± 0.04 [a]	0.02 ± 0.008	0.06 ± 0.05	-	4.53	88.3
Ns 14	2.0 ± 0.3 [a]	0.2 ± 0.1 [a]	0.5 ± 0.2 [b]	0.3 ± 0.2 [a,b]	0.02 ± 0.005 [b]	0.05 ± 0.008 [c]	0.1 ± 0.005 [c]	0.03 ± 0.06	0.06 ± 0.008 [a]	0.4 ± 0.06 [c]	0.03 ± 0.01	0.1 ± 0.09	0.07 ± 0.05	3.86	77.7
Yu 7803	1.6 ± 0.4 [a]	0.1 ± 0.2 [a]	0.5 ± 0.1 [b]	0.5 ± 0.2 [a,b]	0.02 ± 0.005 [b]	0.02 ± 0.005 [b]	-	0.04 ± 0.01	0.2 ± 0.1 [b]	0.2 ± 0.05 [a]	-	0.09 ± 0.06	0.08 ± 0.06	3.35	80.6
Ys 10	2.1 ± 0.6 a	0.7 ± 0.2 b	0.8 ± 0.2 c	0.6 ± 0.2 b	0.03 ± 0.002 c	0.01 ± 0.002 a	-	-	0.2 ± 0.1 b	0.3 ± 0.1	-	0.2 ± 0.1	0.09 ± 0.03	5.03	83.5
Bd 2	2.3 ± 0.4 b	0.4 ± 0.2 a	0.5 ± 0.2 b	0.5 ± 0.2 a,b	0.05 ± 0.006 d	0.02 ± 0.001 b	-	0.03 ± 0.008	0.3 ± 0.1 b	0.5 ± 0.2 b,c	0.01 ± 0.01	-	0.05 ± 0.02	4.66	79.4

Values (mean ± SD, $n = 3$) with no letters in common are significantly different ($p < 0.05$) within a column. "-" means the corresponding compound is not detected. The four main individual phenolic compounds are ChA, Rut, BeA and Ast. Abbreviations: ChA, Chlorogenic Acid; Rut, Rutin; BeA, Benzoic acid; Ast, Astragalin; GeA, Gentianic Acid; Cat, Catechin; VaA, Vanillic Acid; CaA, Caffeic Acid; SyA, Syringic Acid; Epi, Epicatichin; GaA, Gallic Acid; Hyp, Hyperoside; Que, Quercetin.

© 2012 by the authors; licensee MDPI, Basel, Switzerland. This article is an open access article distributed under the terms and conditions of the Creative Commons Attribution license (http://creativecommons.org/licenses/by/3.0/).
